# Supplementary material for: Comparison of anterior nares CT values in asymptomatic and symptomatic individuals diagnosed with SARS-CoV-2 in a university screening program
Source: PLoS One. 2022 Jul 13;17(7):e0270694. doi: 10.1371/journal.pone.0270694 (PMC9278773; doi:10.1371/journal.pone.0270694)
Supplement: S1 Table — (DOCX) [file pone.0270694.s001.docx]

**S1 Table. RNA Polymerase cycle threshold values by month over the course of the study.**

|  | **RP median** | **RP IQR** |
| --- | --- | --- |
| **Total (n= 1633)** | 25.4 | 22.8- 28.1 |
| **Month (n)^a^** |  |  |
| August (n=52) | 24.3 | 22.1- 28.0 |
| September (n=58) | 24.1 | 21.8-27.4 |
| October (n=113) | 25.8 | 23.2-28.6 |
| November (n=231) | 26.2 | 23.5- 28.9 |
| December (n=297) | 26.2 | 23.1- 28.5 |
| January (n=428) | 25.1 | 22.8- 27.8 |
| February (n=303) | 25.0 | 22.6- 27.8 |
| March (n=151) | 24.8 | 22.5- 27.2 |
|  |  |  |

^a^Number of positive tests at BU for each month, from August 2020 to March 2021; March 2021 only has data through March 16^th^.
