# Supplementary material for: Comparison of anterior nares CT values in asymptomatic and symptomatic individuals diagnosed with SARS-CoV-2 in a university screening program
Source: PLoS One. 2022 Jul 13;17(7):e0270694. doi: 10.1371/journal.pone.0270694 (PMC9278773; doi:10.1371/journal.pone.0270694)
Supplement: S2 Table — (DOCX) [file pone.0270694.s002.docx]

**S2 Table. General C_T_ Trends of N2 target**

| **Whole data set with N2 amplified (n=1508)** | **Total** | **Asymptomatic^a^**  **(n= 292)** | **Symptomatic^b^**  **(n= 499)** | **Presymptomatic^c^**  **(n= 717)** |
| --- | --- | --- | --- | --- |
| **Total**  (median,  (Q1- Q3)) | 25.5 (19.4- 32.6) | 30.0* (23.1- 35.7) | 21.4* (17.3- 28.9) | 26.6* (20.1- 32.8) |
| **By Age** (median, (Q1- Q3)) | | | | |
| <20 years (n=476) | 25.3 (19.3- 32.9) | 29.9* (22.1- 35.3) | 21.4* (16.6- 28.3) | 27.0* (20.7- 33.0) |
| 21-25 years  (n= 518) | 26.0 (19.5- 33.1) | 32.0* (26.0- 36.2) | 21.4* (17.7- 28.7) | 27.7* (20.5- 33.2) |
| 26-30 years (n= 160) | 26.1 (19.0- 32.7) | 32.4* (27.2- 36.2) | 21.4* (17.5- 28.7) | 27.6* (20.2- 32.7) |
| >31 years (n= 354) | 25.0 (19.6- 31.3) | 27.2* (22.1- 34.7) | 22.4* (18.9- 29.4) | 25.2* (19.6- 31.0) |
| **By School Affiliation** (median, (Q1- Q3)) | | | | |
|  | **Total** | **Asymptomatic**  **(n= 201)** | **Symptomatic**  **(n= 431)** | **Presymptomatic**  **(n= 479)** |
| **Students**  (n= 1111) | 25.7 (19.3- 33.1) | 30.4* (23.8- 35.7) | 21.5* (17.3- 29.0) | 27.5* (20.5- 33.3) |
|  | **Total** | **Asymptomatic**  **(n= 91)** | **Symptomatic**  **(n= 68)** | **Presymptomatic**  **(n= 238)** |
| **Employees**  (n= 397) | 25.0 (19.6- 31.4) | 27.8* (22.5- 35.1) | 21.1* (17.2- 26.1) | 25.2* (19.6- 31.1) |

^a^ Individuals who did not experience any of the monitored symptoms over infection course.

^b^ Individuals who were experiencing symptoms before testing positive.

^c^ Individuals who developed symptoms the day of or days after positive test.

* p-value is <0.001 using a Kruskal-Wallis rank sum test to compare C_T_ values across symptom categories of asymptomatic, symptomatic, and presymptomatic for each variable; alpha=0.05 used to assess any significant difference between median C_T_ values.
