# Supplementary material for: Comparison of anterior nares CT values in asymptomatic and symptomatic individuals diagnosed with SARS-CoV-2 in a university screening program
Source: PLoS One. 2022 Jul 13;17(7):e0270694. doi: 10.1371/journal.pone.0270694 (PMC9278773; doi:10.1371/journal.pone.0270694)
Supplement: S3 Table — (DOCX) [file pone.0270694.s003.docx]

**S3 Table. N2 C_T_ values by age group**

|  | N2 (median, IQR (Q1-Q3)) | | | |  |
| --- | --- | --- | --- | --- | --- |
| **Age Groups (years)** | **<20** | **21-25** | **26-30** | **> 31** | **p-value^a^** |
| Asymptomatic | 29.9 (22.1- 35.3) | 32.0 (26.0- 36.2)) | 32.4 (27.2- 36.2) | 27.2 (22.1- 34.7) | 0.054 |
| Symptomatic | 21.4 (16.6- 28.3) | 21.4 (17.7- 28.7) | 21.4 (17.5- 28.7) | 22.4 (18.9- 29.4) | 0.89 |
| Presymptomatic | 27.0 (20.7- 33.0) | 27.7 (20.5- 33.2) | 27.6 (20.2- 32.7) | 25.2 (19.6- 31.0) | 0.13 |

^a^ Kruskal-Wallis rank sum tests were conducted for the three symptom groups to assess any difference between Ct values across age categories.

* significant at p< 0.05
