# Supplementary material for: Comparison of anterior nares CT values in asymptomatic and symptomatic individuals diagnosed with SARS-CoV-2 in a university screening program
Source: PLoS One. 2022 Jul 13;17(7):e0270694. doi: 10.1371/journal.pone.0270694 (PMC9278773; doi:10.1371/journal.pone.0270694)
Supplement: S4 Table — (DOCX) [file pone.0270694.s004.docx]

**S4 Table. Comparison of N1 and N2 cycle threshold values for tests with both or only one target amplified.**

|  | **N1 C_T_ values (median, Q1-Q3)** | | | **N2 C_T_ values (median, Q1-Q3)** | | |
| --- | --- | --- | --- | --- | --- | --- |
|  | **Both targets**  **(n=1432)** | **Only N1**  **(n=125)** | P value^a^ | **Both targets**  **(n=1432)** | **Only N2**  **(n=76)** | P value^a^ |
| **Total (n=1633)** | 24.8  (19.2- 31.6) | 36.2  (34.9- 37.3) | <2.2e-16 | 24.9  (19.2- 31.7) | 37.0  (36.2- 37.9) | <2.2e-16 |
| **Asymptomatic** | 28.1  (22.4-33.4) | 36.3  (32.4- 37.3) | 1.21e-13 | 28.6  (22.3- 33.7) | 37.3  (36.5- 38.2) | 3.12e-16 |
| **Symptomatic** | 21.3  (17.1-28.2) | 36.2  (35.4- 37.1) | 4.22e-13 | 21.4  (17.2- 28.3) | 36.3  (36.1- 37.1) | 3.92e-06 |
| **Presymptomatic** | 25.9  (20.0- 32.2) | 36.3  (34.7- 37.4) | 3.23e-13 | 25.9  (20.0- 32.2) | 36.7  (36.0- 37.8) | 2.93e-16 |
| p-value^b^ | < 2.2e-16 | 0.91 |  | < 2.2e-16 | 0.26 |  |

^a^ 2-sample independent Mann Whitney-U test performed to compare median Ct values between those with both targets versus one target for the overall group (“Total”) within symptom categories.

^b^ A Kruskal Wallis test was performed to compare the median Ct values between symptom categories (asymptomatic, symptomatic, and presymptomatic) within variable categories of those with one versus two targets amplified.
