# Supplementary material for: Comparison of anterior nares CT values in asymptomatic and symptomatic individuals diagnosed with SARS-CoV-2 in a university screening program
Source: PLoS One. 2022 Jul 13;17(7):e0270694. doi: 10.1371/journal.pone.0270694 (PMC9278773; doi:10.1371/journal.pone.0270694)
Supplement: S5 Table — (DOCX) [file pone.0270694.s005.docx]

**S5 Table. Symptom type broken out by classification group and in the student and employee population.**

|  | **Total Population who Ever Experienced Symptoms (n=1276)** | **Symptomatic**  **(n= 521, 40.8%)** | **Pre-symptomatic (n= 755, 59.2%)** | **Students who Ever Experienced Symptoms (n=958, 75.1%)** | **Employees who Ever Experienced Symptoms (n=318, 24.9%)** |
| --- | --- | --- | --- | --- | --- |
| **Proportion of those with symptoms who experienced each symptom (%, n)** | | | | | |
| Nasal Congestion | 73.0% (932/1276) | 78.3% (408/520) | 69.4% (524/754) | 78.2% (749/958) | 57.5% (183/318) |
| Cough | 60.0% (761/1276) | 66.4% (346/520) | 55.0% (415/754) | 60.3% (578/958) | 57.5% (183/318) |
| Fatigue | 59.0% (753/1276) | 65.6% (342/520) | 54.4% (411/754) | 60.8% (582/958) | 53.8% (171/318) |
| Sore Throat | 53.1% (678/1276) | 60.7% (316/520) | 47.9% (362/754) | 57.8% (554/958) | 39.0% (124/318) |
| Loss of Smell or taste | 50.3% (642/1276) | 49.3% (257/520) | 51.0% (385/754) | 51.1% (490/958) | 47.8% (152/318) |
| Headache | 48.7% (621/1276) | 56.4% (294/520) | 43.3% (327/754) | 57.2% (548/958) | 23.0% (73/318) |
| Muscle Ache | 44.0% (561/1276) | 48.0% (250/520) | 41.2% (311/754) | 43.1% (413/958) | 46.5% (148/318) |
| Fever +/- Chills | 40.0% (506/1276) | 42.8% (223/520) | 37.5% (283/754) | 39.2% (376/958) | 40.9% (130/318) |
| Nausea | 13.6% (173/1276) | 14.8% (77/520) | 12.7% (96/754) | 13.6% (130/958) | 13.5% (43/318) |
| Shortness of Breath | 13.5% (172/1276) | 16.1% (84/520) | 11.7% (88/754) | 13.6% (130/958) | 13.2% (42/318) |
| Diarrhea | 12.5% (160/1276) | 14.8% (77/520) | 11.0% (83/754) | 11.5% (110/958) | 15.7% (50/318) |
| Dyspnea on Exertion | 6.9% (88/1276) | 8.3% (43/520) | 6.0% (45/754) | 7.0% (67/958) | 6.6% (21/318) |
| Foot or Skin changes/rash | 4.2% (53/1276) | 5.2% (27/520) | 3.4% (26/754) | 5.0% (48/958) | 1.6% (5/318) |
| Vomiting | 2.1% (27/1276) | 2.7% (14/520) | 1.7% (13/754) | 2.1% (20/958) | 2.2 % (7/318) |
