# Supplementary material for: Comparison of anterior nares CT values in asymptomatic and symptomatic individuals diagnosed with SARS-CoV-2 in a university screening program
Source: PLoS One. 2022 Jul 13;17(7):e0270694. doi: 10.1371/journal.pone.0270694 (PMC9278773; doi:10.1371/journal.pone.0270694)
Supplement: Attachment — Submitted filename: Response to Reviewers 04142022.docx [file pone.0270694.s008.docx]

14 April 2022

Dear Dr. Ito,

Thank you for your consideration and the opportunity to revise and resubmit our publication, “**Comparison of Anterior Nares C_T_ Values in Asymptomatic and Symptomatic Individuals Diagnosed with SARS-CoV-2 in a University Screening Program,”** for potential publication in *PLoS ONE*. We have read and considered all the reviewers’ comments, and below respond to them in order.

First, I would like to thank you for considering our initial response in email dated March 22, 2022. In that email we responded to your comment that, “The results for the N1 and N2 genes were so similar, and thus it [may not be] necessary to show them separately for every comparison.” While we agree that keeping both N1 and N2 separate for each comparison is a bit cumbersome, we had a scientific reason to do so.

We agree that the results from N1 and N2 are similar; however, showing only one target will eliminate from our analysis samples that were only positive for one target. Our PCR test is different than many others in that we do not require that both targets be positive to declare a positive result. We can revise, but we feel that some interesting data at lower viral loads (high C_T_) values will be missed if we do that.

Based on your response from March 24, 2022, we will revise the figures to put the N2 panels into the supplementary data for clarity but will include the N1 and N2 data in all tables to represent the full dataset. We have made those changes here.

Further comments from the journal and the reviewers are enumerated and addressed below. Our responses are in bold, and when appropriate are marked on the “tracked changes” copy of the revision.

**Journal Requirements:**

1. Please ensure that your manuscript meets PLOS ONE's style requirements, including those for file naming. The PLOS ONE style templates can be found at 
<https://journals.plos.org/plosone/s/file?id=wjVg/PLOSOne_formatting_sample_main_body.pdf> and 
<https://journals.plos.org/plosone/s/file?id=ba62/PLOSOne_formatting_sample_title_authors_affiliations.pdf>.

2. Thank you for stating the following in the Competing Interests section: 
[I have read the journal's policy and the authors of this manuscript have the following competing interests: Dr. Klapperich is a co-founder of Biosens8, Inc.]

Please confirm that this does not alter your adherence to all PLOS ONE policies on sharing data and materials, by including the following statement: "This does not alter our adherence to PLOS ONE policies on sharing data and materials.” **Thank you, we have included this information in the revised submission under the “comments” section.**

3. We note that you have stated that you will provide repository information for your data at acceptance. Should your manuscript be accepted for publication, we will hold it until you provide the relevant accession numbers or DOIs necessary to access your data. If you wish to make changes to your Data Availability statement, please describe these changes in your cover letter and we will update your Data Availability statement to reflect the information you provide. **We will submit the raw data to figshare upon acceptance. The data will consist of a .csv file with deidentified data points.**

4. We note that you have included the phrase “data not shown” in your manuscript. Unfortunately, this does not meet our data sharing requirements. PLOS does not permit references to inaccessible data. We require that authors provide all relevant data within the paper, Supporting Information files, or in an acceptable, public repository. Please add a citation to support this phrase or upload the data that corresponds with these findings to a stable repository (such as Figshare or Dryad) and provide and URLs, DOIs, or accession numbers that may be used to access these data. Or, if the data are not a core part of the research being presented in your study, we ask that you remove the phrase that refers to these data. **Thank you,** **we have added that all sequence data from Boston University has been uploaded to GISAID, https://www.gisaid.org/. The phrase “data not shown” has been removed.**

5. Please ensure that you refer to Figure 2 in your text as, if accepted, production will need this reference to link the reader to the figure. **Thank you for pointing out our oversight, we have included the Figure 2 reference in the text.**

**Reviewer #1:**

This is a valuable paper with results that will be of interest to decision makers interpreting anterior nares PCR results for COVID-19 testing. Overall, I found the paper to be well written and the study approach and findings to be clearly explained.

In addition to the specific comments below, I would offer the following general comments.

**Comment 1:** The results for the N1 and N2 genes were so similar, I wonder if it is necessary to show them separately for every comparison. The information on results with only one of the two genes detected was interesting, but otherwise, it seems like you combine them in some way (e.g., take midpoint) or just show the analysis for one gene and say the other led to the same conclusions. **Thank you, while we agree that most of the data for N1 and N2 are similar, at low viral loads they are often not, so we have left in both genes due to the reasons discussed above.**

**Comment 2:** The authors could reduce the number of significant digits throughout the paper. Though common to show tenths for percentages, it does tend to imply more precision than is warranted and makes the text and tables harder to read. Also consider the same comment for p-values, e.g., Table 3 don’t need to be shown to 4 digits. **We agree, and we have edited the tables where necessary.**

**Comment 3:** Finding employees were more likely to report no symptoms than students was an interesting result in light of the conventional wisdom that older age groups tend to be more severely affected. Please comment on the discussion on whether this might be due to testing frequency or other factors and compare your results to analogous comparisons from other studies if available. **In our population, students are much more likely to live and work on campus and to live in cohort living situations. It is possible they were exposed more often and for longer periods of time as a result. We know that more students were positive during this time period than staff.**

**Comment 4: Title:** Because viral loads were not measure directly, I would recommend changing to “Comparison of Anterior Nares Ct Values in Asymptomatic …..” **We have made the suggested change.**

**Comment 5: Abstract:** “gold standard method” PCR based on various sample types is certainly the most widespread testing method. I hesitate a bit to call it the gold standard. While it is very sensitive for detecting current or past presence of virus, it is not specific for detection of the infectious period. Also, the performance of PCR of course depends on sample source. **We have deleted this sentence from the abstract.**

**Comment 6:** We collected the N1 and N2 Ct values… I would say collected N1 and N2 target gene Ct values in the first usage. **Added as requested.**

**Comment 7:** “n=931, 1276” should be 932/1276. **Correction made.**

**Comment 8: Introduction:** consider use of gold standard as noted above. **Edited.**

**Comment 9:** **Methods:** “determined to be a false positive by the clinical team” What were the criteria for that determination? **In our cohort, there were individuals working in laboratories that were using amplified genetic material from SARS-CoV-2. People who tested positive and were not truly infected were identified by sequencing and clinical determination and were excluded from this study.**

**Comment 10:** “Symptomatic patients showed symptoms before the day of testing” I found it unclear whether symptoms prior to testing were self-reported contemporaneously through a questionnaire or determined retrospectively through the clinical interview. **Edited to clarify that the symptomatic people presented with symptoms as per a questionnaire.**

**Comment 11:** Results: For discussion, [h]ow did demographic data of the study population compare with the student body and employee demographics? **The study period covers a time over which our population was changing significantly (July 2020 to March 2021). In short, about ¼ of our students were returned to campus between summer and fall and then a larger number returned in person after the break in January. We have not been able to track the demographics in a useful format, and we have not added them to the manuscript**

**Comment 12:** “with asymptomatic cases showing the lowest viral load as expected” suggest deleting “as expected” when presenting results. **Deleted**.

**Comment 13:** Define “staff affiliates” **Deleted “affiliates” for clarity.**

**Comment 14:** Remined >> remained **Fixed.**

**Comment 15:** Were any hospitalizations reported? **During the study period, among students, there were no known hospitalizations due to COVID-19. Among employees, there were 6 known hospitalizations due to COVID-19. We have added this information to the discussion.**

**Comment 16: Discussion:**

“every interview for symptom reporting was conducted by a trained health professional” See note on pretesting symptom reporting above. Also, did interviewers or the respondents have access to Ct values prior to the interview? **See above for comment on attestation questionnaire.** **No, clinical staff did not in general have access to CT values unless an amplicon contamination case was suspected.**

**Comment 17: Figures and Tables** Need to define parenthetic ranges in the tables. **Added.**

**Comment 18:** Table 3: It is not clear which comparisons the p-values apply to. **Edited.**

Sincerely,

Catherine Klapperich, Ph.D.
